# EWET: Data collection and interface for the genetic analysis of *Echinococcus multilocularis* based on EmsB microsatellite

**DOI:** 10.1371/journal.pone.0183849

**Published:** 2017-10-03

**Authors:** Jenny Knapp, Sylvie Damy, Jonathan Brillaud, Jean-Daniel Tissot, Jérémy Navion, Raphael Mélior, Eve Afonso, Vanessa Hormaz, Bruno Gottstein, Gérald Umhang, Adriano Casulli, Frédéric Dadeau, Laurence Millon, Francis Raoul

**Affiliations:** 1 Laboratoire Chrono-environnement UMR CNRS 6249, University Bourgogne Franche-Comté, Besançon, France; 2 UMS 3245 OSU THETA Bourgogne Franche-Comté, Observatory of Besançon, Besançon, France; 3 Institut de Parasitologie de Bern, Bern, Switzerland; 4 Anses Nancy laboratory for Rabies and Wildlife, National Reference Laboratory for *Echinococcus* spp., Wildlife Surveillance and Eco-epidemiology unit, Technopole Agricole et Vétérinaire, Malzéville, France; 5 WHO Collaborating Centre for the epidemiology, detection and control of cystic and alveolar echinococcosis, Istituto Superiore di Sanità, Rome, Italy; 6 European Reference Laboratory for Parasites, Istituto Superiore di Sanità, Rome, Italy; 7 University Bourgogne Franche-Comté FEMTO-ST Institute/CNRS, Besançon, France; Albert Einstein College of Medicine, UNITED STATES

## Abstract

Evolution and dispersion history on Earth of organisms can best be studied through biological markers in molecular epidemiological studies. The biological diversity of the cestode *Echinococcus multilocularis* was investigated in different cladistic approaches. First the morphological aspects were explored in connection with its ecology. More recently, molecular aspects were investigated to better understand the nature of the variations observed among isolates. The study of the tandemly repeated multilocus microsatellite EmsB allowed us to attain a high genetic diversity level where other classic markers have failed. Since 2006, EmsB data have been collected on specimens from various endemic foci of the parasite in Europe (in historic and newly endemic areas), Asia (China, Japan and Kyrgyzstan), and North America (Canada and Alaska). Biological data on the isolates and metadata were also recorded (e.g. host, geographical location, EmsB analysis, citation in the literature). In order to make available the data set of 1,166 isolates from classic and aberrant domestic and wild animal hosts (larval lesions and adult worms) and from human origin, an open web access interface, developed in PHP, and connected to a PostgreSQL database, was developed in the EmsB Website for the *Echinococcus* Typing (EWET) project. It allows researchers to access data collection, perform genetic analyses online (e.g. defining the genetic distance between their own samples and the samples in the database), consult distribution maps of EmsB profiles, and record and share their new EmsB genotyping data. In order to standardize the EmsB analyses performed in the different laboratories throughout the world, a calibrator was developed. The final aim of this project was to gather and arrange available data to permit to better understand the dispersion and transmission patterns of the parasite among definitive and intermediate hosts, in order to organize control strategies on the ground.

## Introduction

The description of diversity among isolates for a given species is a major field of investigation for the understanding of its past and present history on Earth. This kind of data collection needs to be shared among the scientific community working in this field, to ensure experience and knowledge is brought together and thus enhanced. The GenBank sequence database is the best example of an open access genetic and proteomic data source (http://www.ncbi.nlm.nih.gov/genbank/). Dedicated databases are also available on more focused topics in the field of biology, such as the North-East India microbial database NEMiD [1]. This database gathers biological characteristics, sampling details and molecular data aspects relating to soil organisms. The InSatDb is a database putting together annotated microsatellite targets of five fully sequenced insects [2]. From this database, the Web interface allows us, *inter alia*, to find genotyping targets. In the field of host-parasite relationships, the data about the genetic diversity of a parasite associated with host data such as species identity, geographical location, or the level of severity of the disease induced are more powerful when they are gathered in scalable databases. These data, put together in a common database, are more useful because they provide information about the genetic variations between parasite specimens, the pathogenicity of parasite strains circulating in the environment and, when coupled with Geographical Information Systems (GIS) can depict the spread and emergence of host and parasite strains or genotypes over space and time.

The microsatellite EmsB was described for the first time in the cestode *Echinococcus granulosus* for genotyping studies on this infectious agent [3], causing cystic echinococcosis, one of the most important cestode diseases with a huge public health impact globally [4,5]. In this species, EmsB was validated as a highly polymorphic marker where a huge genetic diversity was described [6]. This high diversity was recently described as being due to the tandemly repeated origin of the microsatellite (about 40 copies of the sequence) located on chromosome 5 of *Echinococcus* spp. [7]. To investigate the polymorphism in *E*. *multilocularis*, the infectious agent of alveolar echinococcosis, EmsB was widely used, especially in European isolates, but also in Asia and North America, and has proved its relevance in the description of genetic diversity in this species, where other markers failed [8–13]. Indeed, this parasite belongs to the Cestode class and the Taeniidae family, including four genera, *Taenia*, *Echinococcus*, *Hydatigera*, and *Versteria* [14], and in a genetic diversity point of view, the *Echinococcus* species appear closely related to one another as a monophyletic group, compared to *Taenia* species, described as a paraphyletic group [15,16]. Within *E*. *multilocularis*, very low diversity was described among geographically distinct isolates based on mitochondrial and nuclear targets [17–19], and presents about 10 times lower nuclear diversity than in *E*. *granulosus* sensu lato (s.l.) [19]. The EmsB marker allowed us to describe a genetic diversity level never reached before, because of the polymorphism among its different loci, studied together in fragment analyses, allowing us to emphasize various profiles at regional, local and micro-local geographical scales [7]. The parasite *E*. *multilocularis* is known to occur in the northern hemisphere, and is described as an emerging or re-emerging pathogen in Europe and Asia, mostly in China [20,21]. The parasite involves mammalian hosts in its life cycle (carnivores and small mammals). In Europe the red fox (*Vulpes vulpes*) is its main definitive host, along with other wild and domestic canid carnivores, and rodent species from the *Arvicolinae* subfamily act as main intermediate hosts. It is rare for humans to act as intermediate hosts, and humans are a dead-end host. The parasite is described in 95% of cases to have a tropism for liver with different levels of lesion severity, but can be described in secondary localization in almost any organ (e.g. the lung, spleen, kidney, bones) [22,23]. In humans, the diagnosis is often late, which can lead to a lethal outcome when evolving lesions remain untreated. Moreover, the infection source and time point in humans is often difficult to trace because of a long asymptomatic development period of the parasite in the liver (around 10 years). The incidence in humans is estimated at 18,200 cases per year worldwide, with 91% of cases occurring in China [20].

The distribution of *E*. *multilocularis* EmsB profiles was studied in Europe and in France in order to build scenarios related to its geographical spread over time from traditional to newly described endemic areas [11,24]. Thus, the genetic diversity description from EmsB studies was relevant to help us understand the origin of distribution and contamination in an endemic area presumably stated as “new” [11]. Indeed, in North Poland the genetic diversity was much lower than in the historical endemic areas, such as Switzerland or France. Common profiles were described in the three countries, which could be explained by the occurrence of founder events from old to newly endemic areas, such as in a mainland-island system [25]. Compared to EmsB, few studies, such as other microsatellite investigations or haplotype network experiment, have been additionally done that could help to further explore the genetic diversity in *E*. *multilocularis*, [26,27]. So far, EmsB genotyping appears to be the most convening marker, because producing raw data is simple using a single PCR followed by a fragment size analysis (FSA) (see S1 File EmsB Guidelines sections I to V). Thanks to geographical mapping of the data set constituted from all previous studies performed on the EmsB genotyping, and using a statistical analysis quantifying genetic distance among isolates, this approach may yield in the future, the potential to trace back the contamination event of a patient. These attempts are, however, partly obscured by the heterogeneous geographical distribution of EmsB-related information and the rather small amount of data considering the large areas of investigation. It is therefore a priority to set-up an open and collaborative information system allowing EmsB profiles to be collected at an international level. The aim of the present paper is to introduce the EmsB database (DB) application, named EWET-DB, as well as the EmsB Website for *Echinococcus* Typing database, that allows us to build a reference collection dedicated to *Echinococcus* genetic data by recording, sharing and providing access to the EmsB data.

## Material and methods

### The EmsB data

EmsB genotyping was performed in different projects in order to describe the genetic diversity of *Echinococcus multilocularis*. First of all, the EU EchinoRisk project (QLK2-CT-2001-01995) aimed to assess the risk related to alveolar echinococcosis in Europe and propose a prevention framework. The first collection of isolates from different hosts (human, rodent, monkey and fox) and from different endemic foci (Europe, Asia and North America) was constituted to assess the genetic discriminative power of the EmsB marker [13]. Secondly, various studies were conducted to better understand the genetic variations of the parasite at different geographical levels through the EmsB data, from a regional [11,24] to a micro-local level [13]. At this latter level, the over-discrimination was avoided because rodents from the same field presented common profiles, and were probably infected by the same fox or the same strain circulating locally. A collection of parasites from foxes necropsied in European countries (Austria, Czech Republic, Estonia, France, Germany, Hungary, Italy, Poland, Slovakia, and Switzerland) was thus constituted to better understand the recent dispersion of the parasite [8,11,12,24,28,29]. The study of the newly endemic region of Svalbard (Norway) by EmsB has led to speculation about the origin of contamination on the island [10]. In that study, the parasite harbored by Arctic foxes was certainly transmitted to the first imported rodents on the island by mining activities until the 1960s, establishing a parasite life cycle due to anthropic events. These findings allowed us, for the purpose of the present study, to describe an important genetic diversity in specimens which were shared by definitive and intermediate hosts.

### Toolbox for EmsB data analyses

In order to use the EmsB data generated by FSA, a new approach was developed. For a given sample, the EmsB data were considered as an EmsB profile. This profile was first normalized to make it suitable for analysis, and for comparison, the genetic distance calculation was performed according to a specific protocol. In the present paper an additional tool was developed, known as the calibrator, to allow us to monitor the control of quality of profiles obtained by different FSA automaton models. This tool was developed to control the quality of EmsB profiles and the process of calculating genetic distance.

#### Profile normalization

From each *E*. *multilocularis* specimen, a PCR was performed to amplify the EmsB targets. The PCR products were labeled during the amplification. Reproducibility was previously tested using different *Taq* Polymerases and was then statistically validated [13]. A FSA was then performed on a sequencer machine, in order to separate the PCR products according to their size in base pairs (bp) and obtain the fluorescence intensity value for each size or peak (see S1 File EmsB Guidelines section VI). First the lowest peak values (under 10% of the highest peak) had to be removed as they were considered as artefacts, and data had to be transformed by normalization of the profiles (for each allele, the fluorescence intensity value was divided by the sum of intensities of the whole peaks retained; see S1 File EmsB Guidelines section VI).

#### Genetic distance calculation

To study *E*. *multilocularis* genetic diversity and spatio-temporal relationships, the evaluation of the genetic distance among samples was performed. Distance calculation was achieved by using hierarchical clustering analysis. First, a distance matrix among isolates was computed based on the Euclidean distance (see S1 File EmsB Guidelines section VII). Then, linkage between isolates was formed using the Unweighted Pair-Group agglomerative Method using Arithmetic mean (UGPMA) and a dendrogram of distance was generated. UGPMA allows an object (EmsB profile) to join a group of objects at the mean of the Euclidean distance between this object and all members of the group [30]. Multiscale bootstrap resampling is used to compute p-values of each group, thereby providing information about the likelihood of each node of the dendrogram, thanks to the pvclust R package [31].

#### EmsB calibrator

In order to control the EmsB quality obtained by the different working groups, a calibrator was designed in the present study. FSA of the labeled EmsB PCR products in the various surveys were performed using different analyzers, e.g. ABI PRISM 310 Genetic Analyzer [7,24,32], Applied Biosystems 3130 Genetic Analyzer [8,11–13], Applied Biosystems 3730 DNA Analyzer [10], Applied Biosystems 3500 Genetic Analyzer [32] (Life Technologies, Foster City, CA), or Beckman CEQ 8000 [3,8,13] (Beckman Coulter, Fullerton, CA). With these different analyzers, a shift in the fragment size reading can be observed because of different labeling dyes used, between amplicons and standard molecular weight, influencing migration of the different components during capillary electrophoresis [7,33].

**Fig 1:**
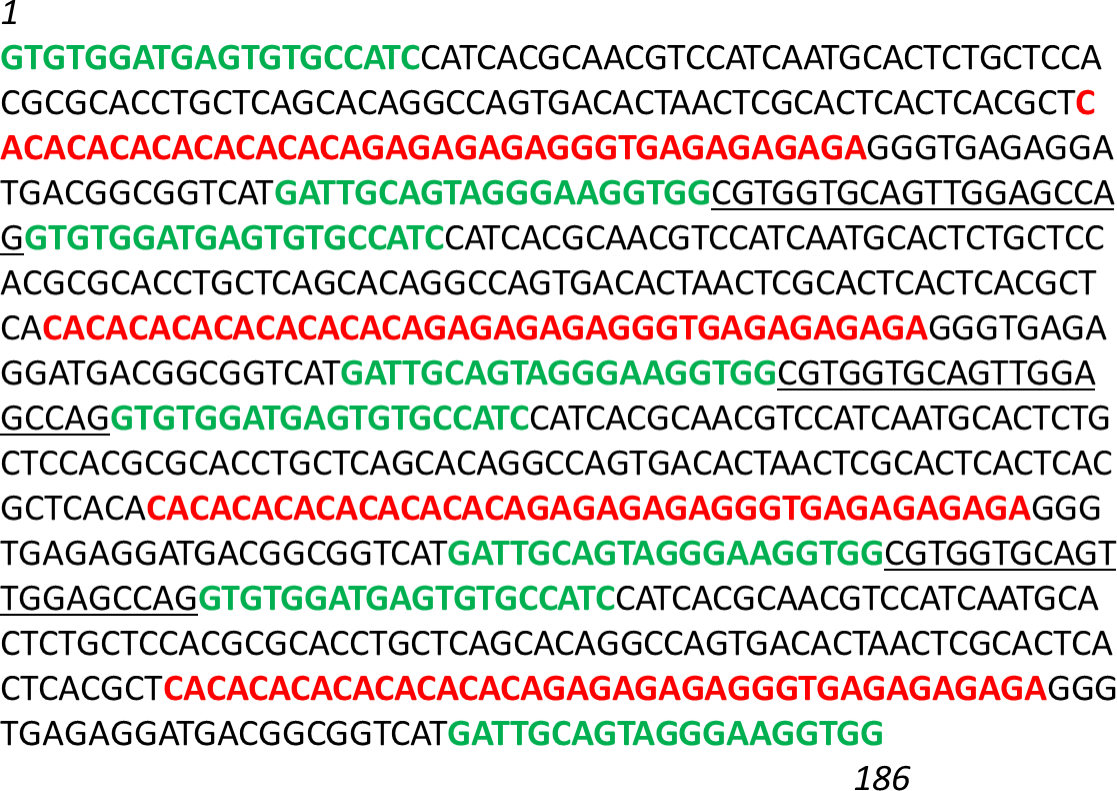
The EmsB calibrator sequence. The sequence contains 4 microsatellite sequences (red) and flanking regions with forward and reverse primers (green) and interspacers (underlined). In order of appearance, sequence 1: 190 bp (with a microsatellite of 40 bp); sequence 2: 192 bp (42 bp); sequence 3: 194 bp (44 bp), sequence 4: 190 bp (40 bp), for a total of 826 bp.

Based on the EmsB sequence (GenBank access code number AY680860.1), a plasmid construction was made with four EmsB microsatellites, corresponding to (CA)_9 to 11_ (GA)_11_ repeats respectively, two microsatellites having the same size (including flanking regions) with 190 bp, a second with 192 bp and the last one with 194 bp. Part of their original flanking regions was conserved from either sides of the microsatellite sequences (Fig 1). From these flanking regions the amplification by PCR is possible with the EmsB A-forward and EmsB C-reverse primers, EmsB A being labeled with a fluorochrome (FAM with ABI automatons) [13]. Thereby the construction of a nucleotide sequence of 826 bp was generated by GeneCust services (Dudelange, Luxembourg). The sequence was inserted in a plasmid pET-11aH6 [34], in the transformed *Escherichia coli* DH5alpha. After culture, purification of the plasmids was performed with the QIAfilter Plasmid Midi kit (Qiagen, Hilden, Germany), as recommended by the manufacturer. The elution step was performed with Tris-EDTA buffer solution pH 8 (Sigma Aldrich, Saint Louis, MO). A stock solution with a DNA plasmid concentration of 3000 ng/μl was obtained and used with a 1:1000 dilution in PCR and stored at -20°C until use.

PCR was performed in a 20 μl reaction mixture containing 3 ng of plasmid DNA, 10 μl of the AmpliTaq Gold PCR Master Mix 2X (0.5 U of enzyme) (Roche, Branchburg, NJ), 0.3 μM of fluorescent forward primer, 5’-labeled specific fluorescent dye, 0.5 μM of classical reverse primers. The PCR amplification was achieved in a thermocycler under the following conditions: an initial denaturation step at 94°C for 5 min, and 25 cycles with denaturation at 94°C for 30 secs, annealing at 60°C for 30 secs, extension at 72°C for 30 secs and a final extension step at 72°C for 5 min. PCR product size was controlled by electrophoresis in 1% agarose gel.

The FSA was performed on the Applied Biosystems 3130 Genetic analyzer (ABI-3130) (Life Technologies, Foster City, CA) and the results were analyzed on GeneMapper 3.7 software.

### EWET-DB to manage EmsB data

**Fig 2:**
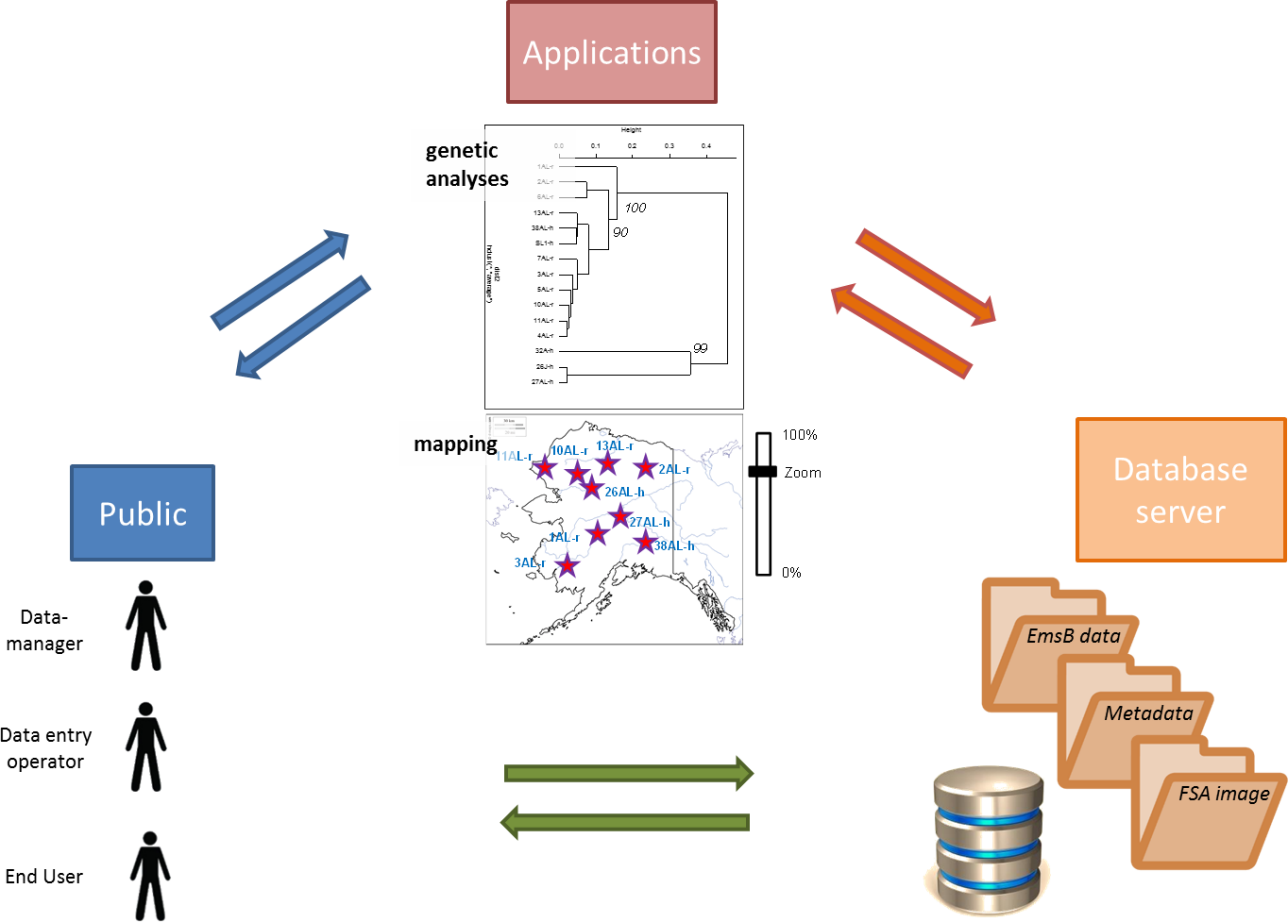
EWET organization and implementation.

In order to manage the gathered EmsB data and their associated metadata in a rigorous and systematic way, a database was developed with an open access web interface (Fig 2).

#### Database characteristics and functionalities

**Fig 3:**
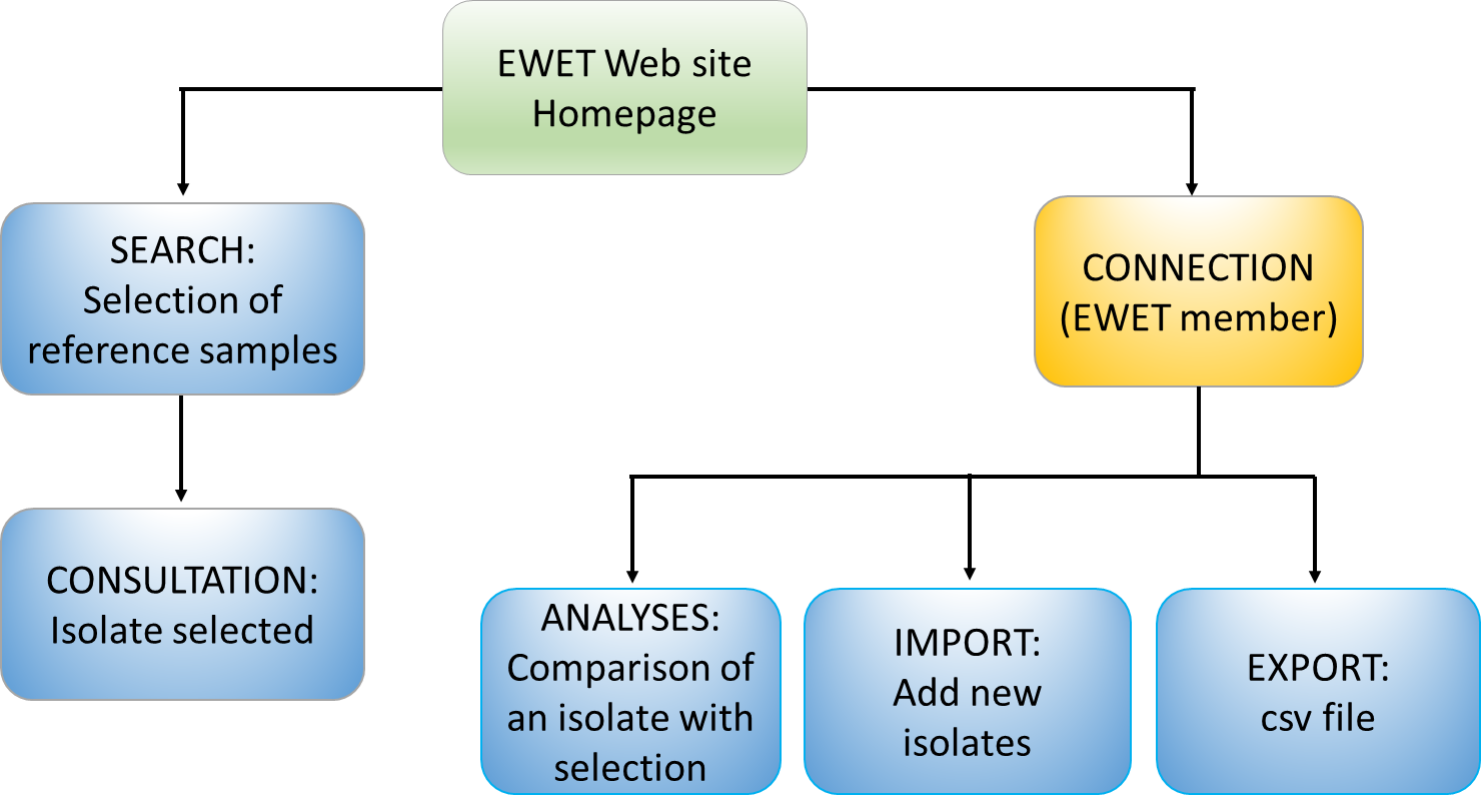
Web interface. The general presentation of the EWET-DB website.

**Fig 4:**
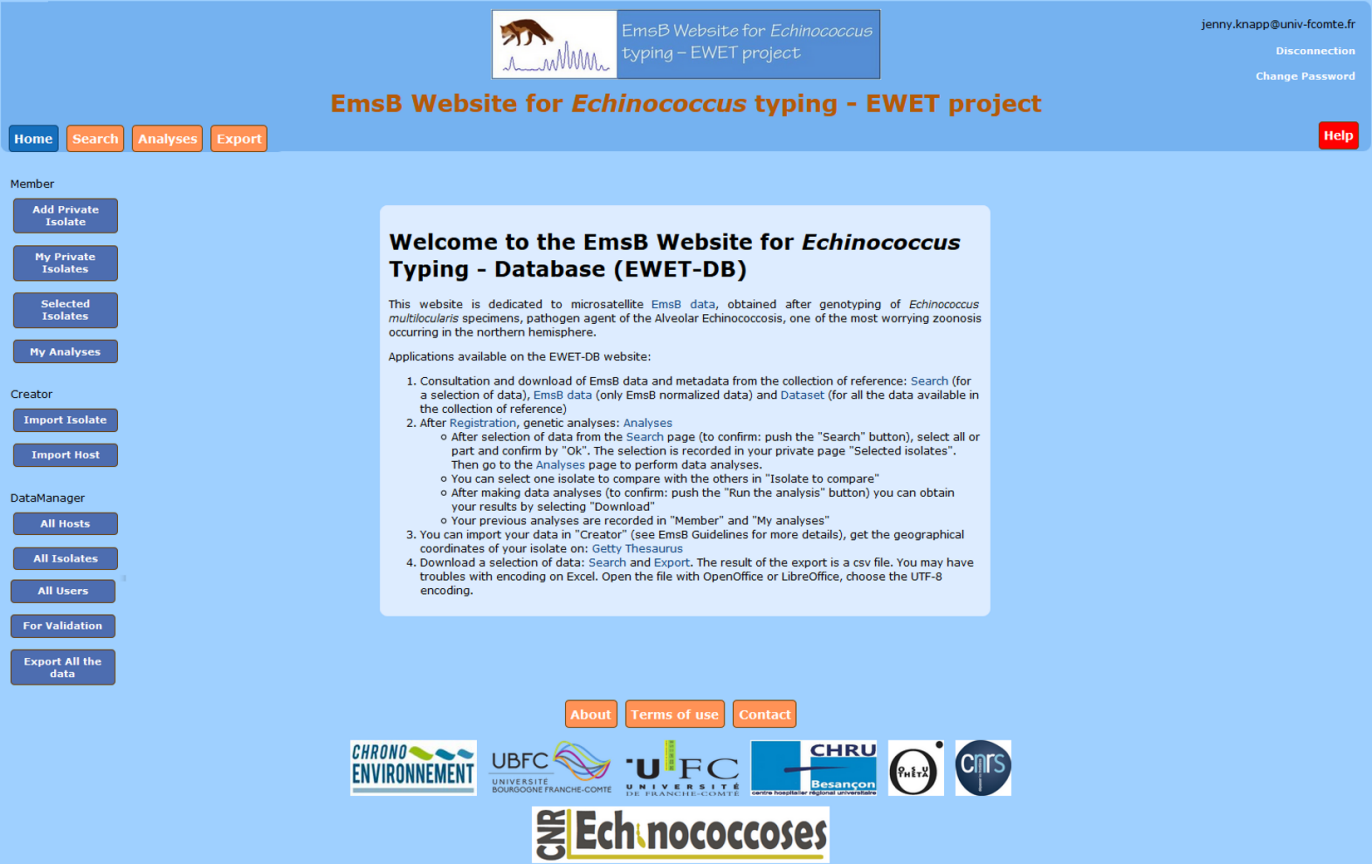
The “home” page of the EWET-DB website.

**Fig 5:**
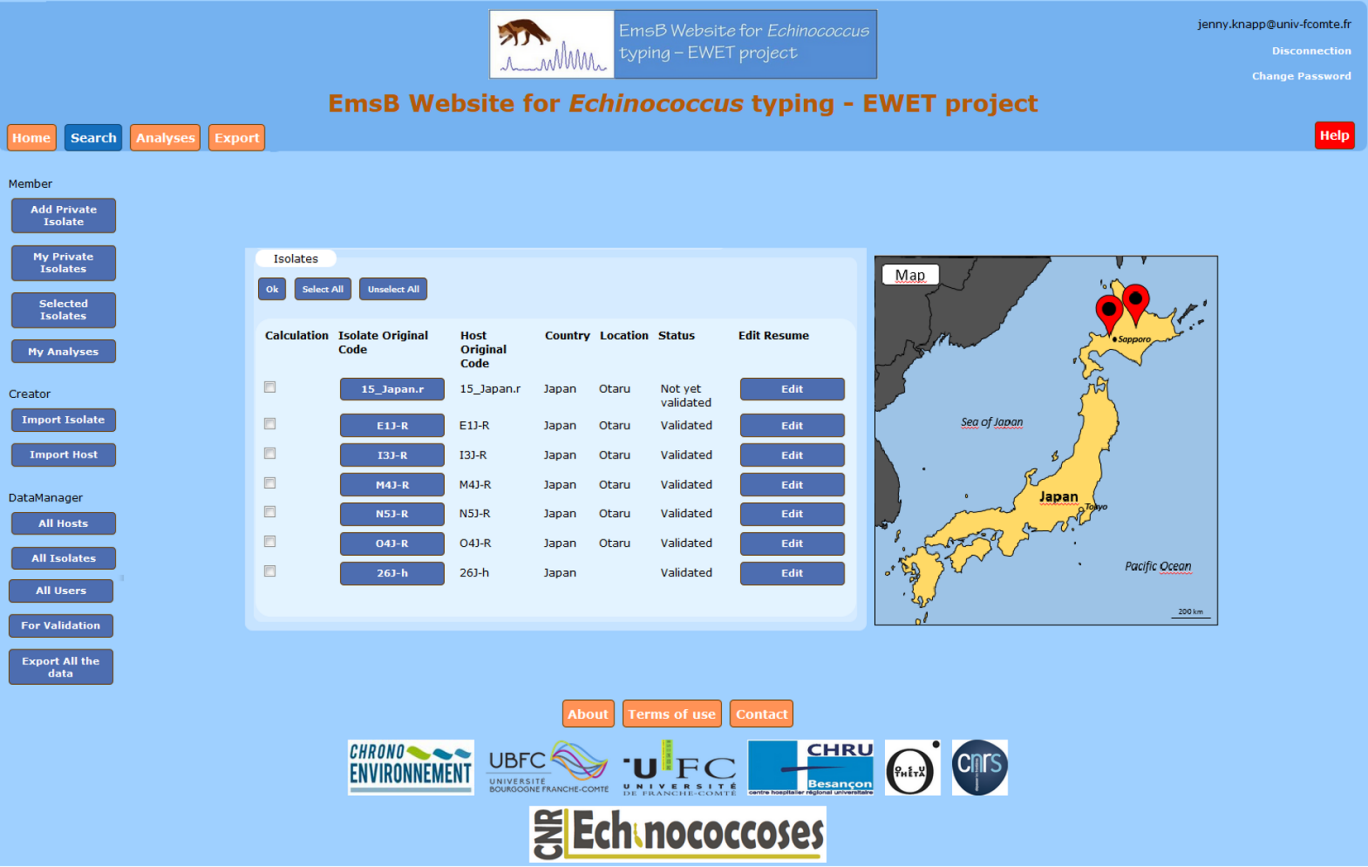
The “search” page of the EWET-DB website. Data sheep and distribution map for selected isolates.

The database was developed in PHP and connected to a PostgreSQL database. The different applications of the EWET web site are presented in Fig 3. The homepage of the EWET application (Fig 4) gives a general introduction to the EmsB microsatellite database. The “search” page can be used to consult isolate data (including EmsB data) and to consult the distribution map from the database using different criteria (host, country, province, year of sampling, etc.) (Fig 5). After registering and selecting isolates, the “analyses” page allows us to calculate genetic distances among samples, generate dendrograms. The results of the analysis can be obtained from the “Download” button. The “export” page allows us to download data from a selection of isolates, and contains the EmsB guidelines providing full details of the microsatellite and the description of the online analyses (S1 File EmsB Guidelines). R scripts are likewise available for users who wish to obtain more details on the analyses or to perform the analysis without the EWET application. EWET members can access and download the EmsB data and the all dataset, and record their isolates once they have registered. They can choose whether they would like their data to be shared or kept private.

#### Data quality

Data recorded in the database were standardized as much as possible by using frameworks recognized by the International community, such as species names, geographical locations and coordinates. To take into account the privacy issues in relation to patients, their GPS location (private geographical location at diagnosis) was made less specific for those consulting the data (at county level).

#### Code assignation

In the previous EmsB studies, a code for each isolate was generated by each laboratory and is essential for recording further investigations between working groups and for archives. However, as well as this original code, we need a unique code format in a common dataset on these samples for standardization purposes.

## Results and discussion

### Data set description

The compilation of published EmsB data allowed us to generate a data set composed of 1166 isolates from adult or larval stage of the parasite *E*. *multilocularis*, from definitive and intermediate hosts (red foxes, domestic dogs, wild and domestic cats, raccoon dogs, humans, rodents (7 species) and monkeys (3 species)), from 16 countries (Europe: Austria [N = 99], Czech Republic [N = 67], Estonia [N = 11], France [N = 559], Germany [N = 91], Italy [N = 17], the Netherlands [N = 2], Norway (Svalbard) [N = 27], Poland [N = 95]; Slovakia [N = 63], Switzerland [N = 108]; North America: United States of America (Alaska) [N = 13], Canada [N = 1]; Asia: Japan [N = 6], Kyrgyzstan [N = 2] and the People’s Republic of China [N = 5]). The origin of the samples are available in the dataset, all samples taken from published works. Approval from an ethical committee or institutional review board was not necessary for these researches, because of the nature of the samples (parasite material).

The EmsB data collected in this data set were collected from analyses performed on four different sequencer machines (Beckman CEQ8000 DNA sequencer (CEQ8000), ABI Prism 3100 Genetic analyzer (ABI-3100), Applied Biosystems 3730 DNA Analyzer (ABI-3730) and Applied Biosystems 3500 Genetic analyzer (ABI-3500)), in four laboratories (Laboratory of Biochemistry, Besançon University Hospital, France; Laboratory of Genetics, University of Berne, Switzerland; WHO Collaborating Centre for Epidemiology, Detection and Control of Cystic and Alveolar Echinococcosis, Istituto Superiore di Sanità, Rome, Italy and National Reference Laboratory for *Echinococcus* spp., Anses Laboratory, Nancy, France). Analyses previously performed on an ABI-310 machine [24] were performed again on an ABI-3500 machine, as recommended by the manufacturer, to compare data with other versions. Using the electrophoregram pictures available for the EmsB profiles it is possible to visually compare samples, and the original intensity of each locus recorded provides data quality control. Pictures, original fluorescence intensities and details of the geographical location of samples were gathered from the literature and from computer and paper archives.

### EmsB calibrator

**Fig 6:**
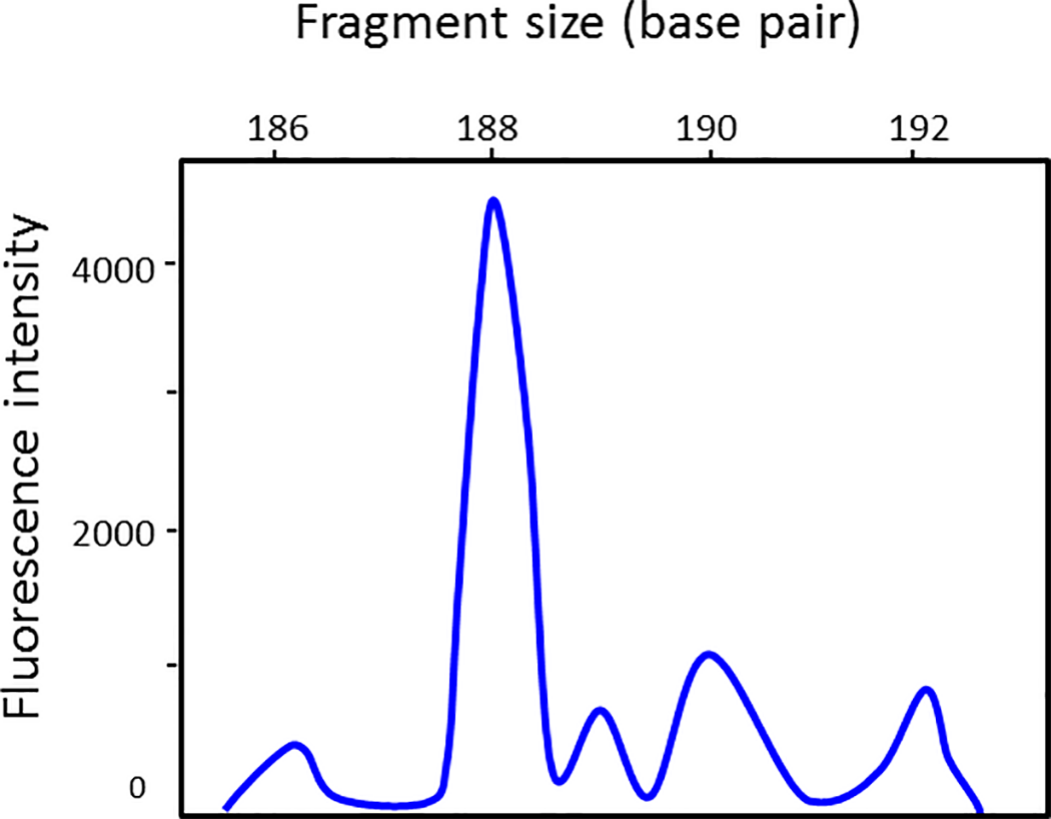
Fragment size analysis of the EmsB calibrator. Data from 3130 Genetic analyzer apparatus.

A nucleotide fragment containing four EmsB microsatellite sequences was generated and included in a plasmid to design a calibrator for all new EmsB fragment size analyses. First, this assay has allowed us to compare the size in bp between the construction and the result of the analysis performed with the automaton. A shift of 2 bp was observed between the electrophoregrams obtained with an ABI-3130 automaton and the expected size in the construction. The same result was previously obtained between electrophoregrams of EmsB profiles for genotyping of isolates and the expected size of the microsatellite in the *E*. *multilocularis* genome [7]. The EmsB-calibrator will be recommended to the different laboratories in order to carry out quality control on the results and authors submitting a new EmsB profile will be asked to use it. Secondly, the calibrator study has allowed us to observe the non-proportionality (from a mathematical point of view) between the number of copies of a given microsatellite size and the fluorescence intensity. With the ABI-3130, the microsatellite in two copies (188 bp) presented a fluorescence intensity that was four-times higher than the second peak (190 bp), and five-times higher than the third peak (192 bp), both representing a microsatellite in one copy (Fig 6). The intensity of fluorescence has to be taken into account in the comparison of EmsB profiles but not in the interpretation of the number of microsatellite copies.

#### Profile normalization and electrophoregram picture

From the published data, original fluorescence intensity values were gathered from archives for part of the data set. For some samples, only the normalized data were available. For all future submissions, the original fluorescence intensity values at each fragment size detected (from 209 to 245 bp) will be requested and the profiles will be automatically normalized with the EWET application. Coupled with the EmsB electrophoregram and calibrator pictures requested, quality control can be achieved by data managers.

### EWET-DB database description

#### The data model

**Fig 7:**
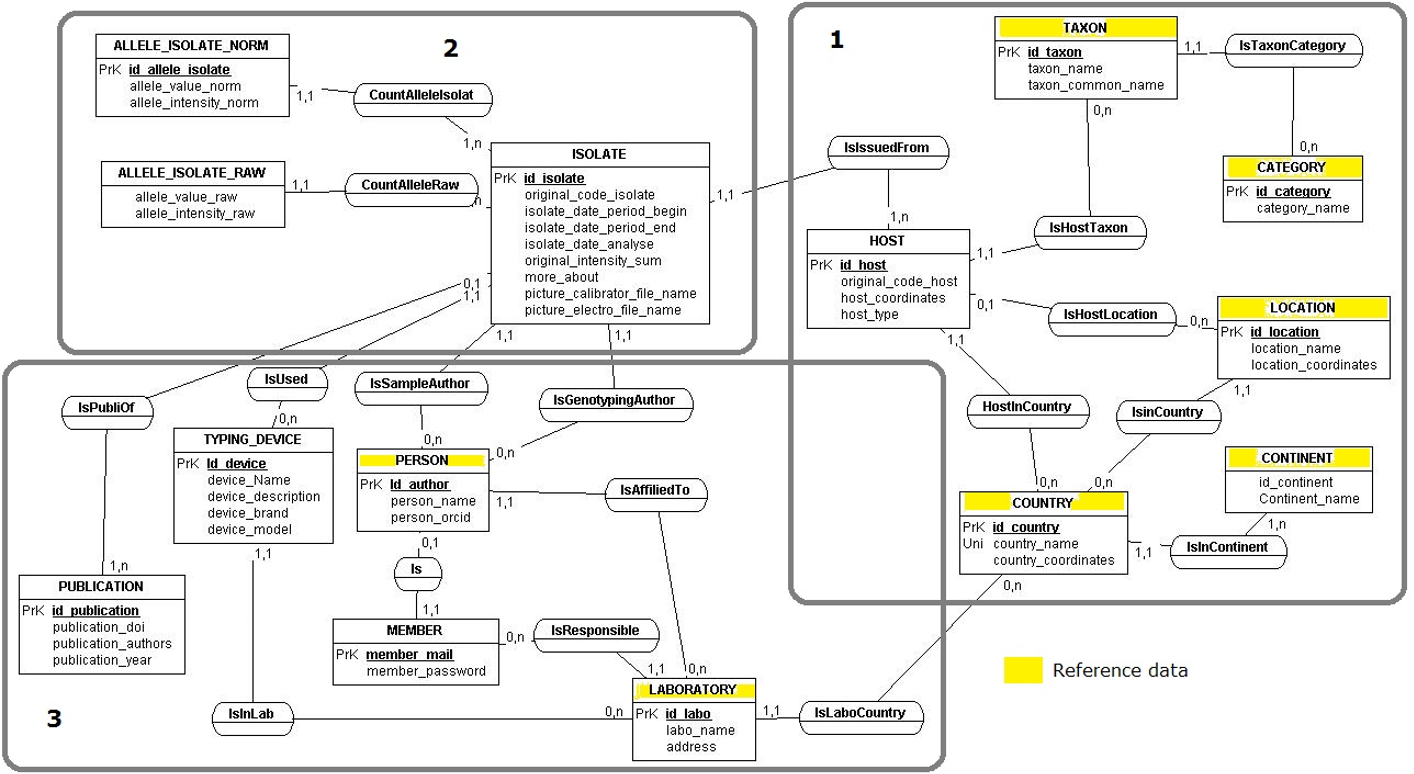
Entity relationship diagram of the EWET database. Zone 1 dedicated to the isolate host, zone 2 dedicated to the isolate, zone 3 provides administrative elements.

The first step was to design the data model (Fig 7) [35]. The isolate is the central entity of the data model. Information about the isolate was gathered from the literature, computer and paper archives, as were the sampling author and sampling date, the host species, the sample location (GPS coordinated, from Getty TGN available online), the author of the FSA, the EmsB fluorescence intensity values (original, normalized data, both or neither), the picture of the EmsB electrophoregram in JPEG format, and the details on automaton used.

The data model consists of three main parts (Fig 7): zone 1 describes the host of the isolate (e.g. taxonomical classification and spatial information); zone 2 was dedicated to the isolate and gave genotyping results; zone 3 provides administrative elements such as genotyping or sampling authors, laboratories, used devices and publications linked to the isolate.

#### Application architecture

A postgreSQL database was created from this model and the existing data was successfully integrated.

The EWET application allows us to centralize and record all the data concerning isolates genotyped for the EmsB microsatellite in a unique database. It makes these data available with remote access for the scientific community (https://ewet-db.univ-fcomte.fr). It ensures the quality of the data and provides easy access to tools for calculating EmsB (distance matrix, dendrogram, comparison, maps), thanks to the dedicated interfaces (Fig 3). Users need to connect to access functionalities like “Analyses” and “Export”.

#### EWET application private isolate and data records

The submission interface offers two functionalities: (i) comparison between the data collection and new data without recording, saved as private isolates (only accessible by the author) and (ii) record new data (for public consultation) in the data collection, saved as new isolates (in the “Creator” part of the Website). For calculation with private isolates, the original fluorescence intensity values are required. The values are automatically normalized and the user is then redirected to the “Analyses” page for further comparisons with the reference collection. For recording, a minimum amount of data is required from the author of new EmsB records. The web application is encoded to allow a submission to be made when the following mandatory items are provided: original sample code, country (level 1), location (level 2), host species type, host category, host name, sampling date, FSA author, EmsB original fluorescence intensity values at each fragment size detected (from 209 to 245 bp), picture of the electrophoregram (JPEG format required), FSA machine used, whether an EmsB-calibrator was used or not, sampling author and publication information. The more data the author provides, the more the information is relevant for the community. The author of the submission and all other authors involved in the sampling and analyses agree to make their data publicly available on the EWET-DB. When the submission is complete, the data enter a quality control process before being published with a unique EWET code on the EWET website.

#### Data quality

The quality control of EmB data was improved in different ways in order to create a reference collection. Data recorded in the present database are standardized as far as possible by using reference lists (Reference data in Fig 7) recognized by the community, such as taxonomic classification according to Wilson and Reeder's Mammal Species of the World online database [21], name of the geographical position and coordinates (decimal degree), obtained thanks to the Getty Thesaurus of Geographic Names (TGN), available online (http://www.getty.edu/research/tools/vocabularies/tgn/), and the name and code of the country from the ISO 3166, the International Standard for country codes, and ORCID code author (http://orcid.org).

The submission of new EmsB data is first controlled by the data manager, who validates the data and sends the corresponding EWET-code to authors. Secondly, the standardization of genotyping data is performed using the EmsB-calibrator developed in the present study. The calibrator must be run with all new EmsB genotyping submissions.

#### Identification of data

The automatic EWET-code "id_isolate" allocates a unique code to identify all EmsB isolates genotyped and allows the authors to pinpoint their own data. Each EmsB sequence receives a code corresponding to a compilation of (i) the order number of registration in the database, (ii) the 3 letters of the ISO Code 3166–1. For example, the EWET-code of an isolate recorded with the value 233 for id_data, isolated in Switzerland, receives the code “EWET-233-CHE”.

The original codes for the isolate and its host, and the codes allocated during registration are recorded in the database.

### Extension to other databases

The EWET-DB contains data about human and animal hosts. For this reason the database can be linked to other information systems such as the EurEchino database (http://cnr-echino-alveolaire-ccoms.univ-fcomte.fr) for epidemiological and medical data about alveolar echinococcosis patients [36], and to a database on small mammal intermediate host communities in France, China and Kyrgyzstan, developed by the authors [FR, University of Bourgogne Franche-Comté, France]. For the specimens studied on additional genetic markers, the GenBank access number could be available in the EWET-DB. With the advent of new generation sequencing, additional genetic data and metadata could be linked to the specimens included in the EWET-DB in the future. Moreover, in the item named “more about”, additional information can be found about each specimen.

## Conclusion

Collecting, organizing and sharing data is fundamental in science, but it is only possible if data are managed and stored efficiently and metadata are made available. Centralizing data is a key step towards valorizing the data generated, and for promoting international collaborative projects. The EWET-DB is dedicated to sharing genetic data about *E*. *multilocularis* focused on the EmsB microsatellite, a powerful marker for genetic studies of the parasite, as well as genetic data associated with information on the parasite host and geographical coordinates. The available online dataset allows researchers to perform genetic and spatial analyses on the reference collection and their own data. The quality control of the EmsB analysis will improve thanks to the calibrator. EmsB data integrated in a database will be in a secure environment, with accurate and controlled information from sampling to genetic treatment. The interoperability of the EWET-DB to other data banks, e.g. with epidemiological and medical databases, will help us to better understand the links between the genetics of the parasite and the occurrence of the disease in humans and animals.

## Supporting information

S1 File EmsB guidelines. Description of the EmsB microsatellite and guide to perform the analysis without the EWET website application.
(PDF)
