# Supplementary material for: EWET: Data collection and interface for the genetic analysis of Echinococcus multilocularis based on EmsB microsatellite
Source: PLoS One. 2017 Oct 3;12(10):e0183849. doi: 10.1371/journal.pone.0183849 (PMC5626424; doi:10.1371/journal.pone.0183849)
Supplement: S1 File — Description of the EmsB microsatellite and guide to perform the analysis without the EWET website application. (PDF) [file pone.0183849.s001.pdf]

EmsB analysis guidelines

Guide to using the EmsB tool and for off-line use

EmsB Website for *Echinococcus* typing – EWET project

Summary

I. Introduction to the EmsB microsatellite 3
II. DNA extraction 3
III. EmsB Primers 4
IV. Amplification by PCR 4
V. Size of polymorphism analysis 4
VI. Example of EmsB electrophoregram and interpretation 4
1. Recording EmsB data on a spreadsheet 5
a) EmsB fragment size 5
b) Fluorescence intensity 5
VII. Euclidean distance calculation, dendrogram and examples for training 6

I. Introduction to the EmsB microsatellite

The EmsB marker is a microsatellite present in about 40 copies in the *Echinococcus multilocularis* genome, located on chromosome 5 [1]. The flanking regions of EmsB are highly conservative, but the microsatellite pattern $(CA)_n(GA)_n$ present a size polymorphism, with independent mutations occurring in the (CA) and the (GA) repetitions (Figure 1). In order to study the microsatellite polymorphism in *E. multilocularis* specimens, a PCR was performed on these targets and primers were designed in the microsatellite flanking regions [2]. The EmsB marker was used for genotyping *E. multilocularis* on different geographical scales, from a micro-local scale to a regional scale [3–8]. These guidelines will allow researchers to use and analyze EmsB data from their own samples, from DNA extraction to genotyping studies.

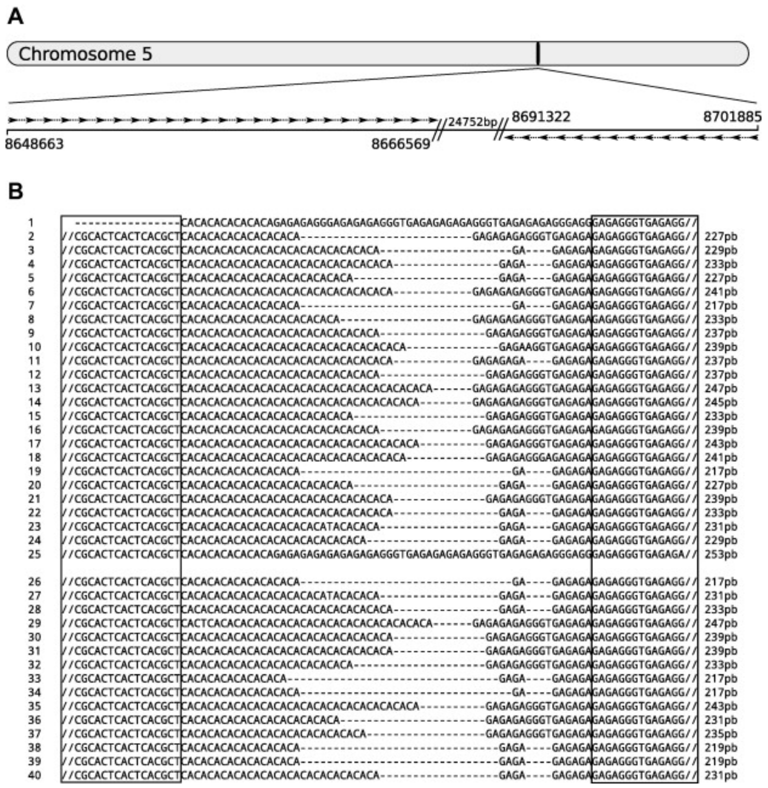

Figure 1. Structure of EmsB microsatellites in *E. multilocularis*. (A) Represents schematic structure on chromosome 5. Arrows indicate one repetition of the microsatellite. Dot points between arrows refer to inter-simple sequence repeats; (B) represents alignment of variable regions of the microsatellite. Sequence number corresponds to the order of repetitions in chromosome 5.

II. DNA extraction

Total genomic DNA is isolated and purified from a tissue sample (unique worm, isolated egg or approx. 50 mg of metacestode), using a DNA extraction kit for tissue. The procedure is carried out according to the manufacturer's protocol. Purified DNA is eluted with 200 µl of elution buffer (provided by the manufacturer) for metacestode samples and unique worm, or 100 µl for eggs, in order to obtain optimal DNA concentrations. The DNA concentration is checked with a spectrophotometer apparatus. The limit of sensitivity was 1 fg of DNA used for EmsB-PCR [2]. Theoretically, DNA purified from one egg can be used as a matrix for PCR. The DNA samples have to be stored at -20°C until use in PCR.

III. EmsB Primers

Primers were designed in the highly conservative flanking region of the microsatellite (Figure 1). The EmsB A primer is 5'-labeled with a fluorochrome (ex. FAM), and is 20-bp long. The EmsB A primer can be ordered as a "modified" oligonucleotide. The EmsB C, 20-bp long primer can be ordered as an "unmodified" primer.

Table 1. EmsB primers: description and reference.

Primer name	Primer sequence	Size of amplicons	Microsatellite repetition	Annealing temp (°C)	Genbank Reference
EmsB A	5'(FAM)-GTGTGGATGAGTGTGCCATC-3'	209-241 bp	$(CA)_n(GA)_n$	60	AY680860
EmsB C	5'-CCACCTTCCCTACTGCAATC-3'				

IV. Amplification by PCR

The EmsB-PCR is performed in a 30 µl reaction mixture containing 50 to 100 ng of DNA, 200 µM of each deoxynucleoside triphosphate, 0.4 µM of fluorescent forward primer, 5-labeled specific fluorescence dye, 0.7 µM of classical reverse primers, and 0.5 U of enzyme, e.g. *Taq* DNA polymerase enzyme associated with the corresponding PCR buffer. The PCR amplification is achieved in a thermocycler under the following conditions: an initial denaturation step at 94°C for 5 min and 30 cycles with denaturation at 94°C for 30 s, annealing 60°C for 30 s, extension at 72°C for 1 min and a final extension step at 72°C for 10 min (minimum). One PCR is enough to obtain an EmsB profile. It is possible (but not essential) to control the size of the PCR products by electrophoresis on 1% agarose gel.

V. Size polymorphism analysis

PCR products are studied in fragment size analysis. To assess the polymorphism of size, an automatic sequencer can be used, such as ABI Prism 3100 or 3500 automatic sequencer (Life Technologies, Foster City, CA) or Beckman CEQ 8000 (Beckman Coulter, Fullerton, CA). A molecular-weight size marker is used to specify the size of the PCR fragments. The fluorescence signal generated by the labeled primer is detected by colorimetric reading. Correspondences are established to assess the size of the amplified fragments using dedicated software (e.g. Genotyper 3.7 for the ABI apparatus or Genetic Analysis System 8.0.52 for the Beckman apparatus).

VI. Example of EmsB electrophoregram and interpretation

After the fragment size analysis is performed, an EmsB electrophoregram is obtained (Figure 2). We can observe size standard (1) and a series of peaks (2) with different sizes (from 209 to 241 bp observed in *E. multilocularis* samples from endemic regions worldwide). The height of peaks is different from one peak to another and refer to the number of EmsB fragment copies for a given size. The size and fluorescence intensity for each peak are recorded in a spreadsheet (Table 2).

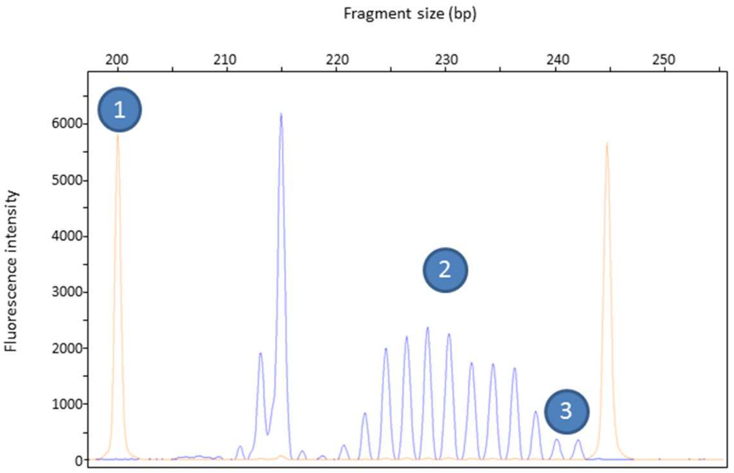

Figure 2. Electrophoregram of EmsB-PCR products performed on a 3100 automatic sequencer (Life Technologies, Foster City, CA). (1): size standard in orange (GS 5000 Gene Scan 500 LYZ), (2): the EmsB fragments classified by size in blue (in base pair) and (3) peaks under 10% of the highest peak to be removed from the analysis.

1. Recording EmsB data on a spreadsheet:

a) EmsB fragment size

On the electrophoregram, the size of the fragments has to be adjusted (see the “raw peak size” and “adjusted peak size” lines in Table 2 - Step 1). Because the EmsB variations are due to the addition or suppression of 2 nucleotides (CA) and (GA), the minimum distance between two peaks is 2 bp.

b) Fluorescence intensity

For each peak, the fluorescence intensity (FI) is recorded (Table 2 – Step 2). This intensity depends on the initial quantity of DNA used for the PCR. First the lowest peak values (under 10% of the highest peak) have to be removed. Second the FI values are thus normalized: for each peak the FI is divided by the sum of the entire FI for a given sample (e.g. for the peak at 215 bp ➔ 6185/21797 = 0.28). The sum of the normalized values is equal to 1. The distance between samples will be calculated according to these normalized values. NB. substitute coma by point. Fragment sizes and normalized FI (Table 2 – Step 3) are saved in a text file in tab format (*.txt) (Figure 3).

Table 2. Information on an EmsB fragment size analysis to be recorded for genotyping studies

Step 1: Adjust the peak size

Raw peak size (size standard)	214.99	216.92	218.75	220.78	222.72	224.56	226.51	228.45	230.41	232.46	234.42	236.39	238.35	240.38	242.32
Adjusted peak size	215	217	219	221	223	225	227	229	231	233	235	237	239	241	243

Step 2: Normalize the fluorescence intensity

Adjusted peak size	215	217	219	221	223	225	227	229	231	233	235	237	239	241	243	
Original Fluorescence intensity (FI)	6185	0	0	300	831	1985	2210	2368	2252	1741	1716	1642	867	455	450	Sum
FI without peak 10%	6185	0	0	0	831	1985	2210	2368	2252	1741	1716	1642	867	0	0	21797
Normalized FI values	0.28	0	0	0	0.038	0.091	0.101	0.108	0.103	0.079	0.078	0.075	0.039	0	0	1

Step 3: Values retained for calculation

Adjusted peak size	215	217	219	221	223	225	227	229	231	233	235	237	239
Normalized FI values	0.28	0	0	0	0.038	0.091	0.101	0.108	0.103	0.079	0.078	0.075	0.039

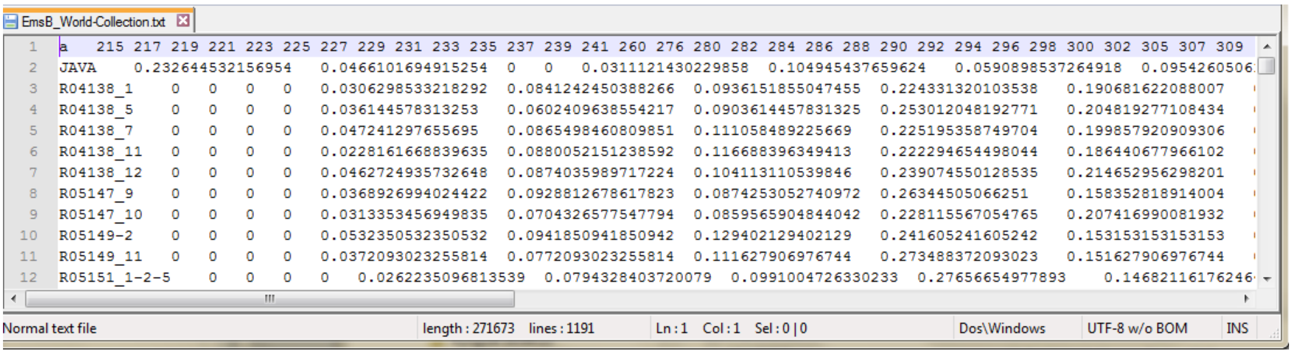

EmsB_World-Collection.txt

```
a    215 217 219 221 223 225 227 229 231 233 235 237 239 241 260 276 280 282 284 286 288 290 292 294 296 298 300 302 305 307 309
JAVA    0.232644532156954    0.0466101694915254    0    0    0.0311121430229858    0.104945437659624    0.0590898537264918    0.0954260506
R04138_1    0    0    0    0    0.0306298533218292    0.0841242450388266    0.0936151855047455    0.224331320103538    0.190681622088007
R04138_5    0    0    0    0    0.036144578313253    0.0602409638554217    0.0903614457831325    0.253012048192771    0.204819277108434
R04138_7    0    0    0    0    0.047241297655695    0.0865498460809851    0.111058489225669    0.225195358749704    0.199857920909306
R04138_11    0    0    0    0    0.0228161668839635    0.0880052151238592    0.116688396349413    0.222294654498044    0.186440677966102
R04138_12    0    0    0    0    0.0462724935732648    0.0874035989717224    0.104113110539846    0.239074550128535    0.214652956298201
R05147_9    0    0    0    0    0.0368926994024422    0.0928812678617823    0.0874253052740972    0.2634450506251    0.158352818914004
R05147_10    0    0    0    0    0.0313353456949835    0.0704326577547794    0.0859565904844042    0.228115567054765    0.207416990081932
R05149-2    0    0    0    0    0.0532350532350532    0.0941850941850942    0.129402129402129    0.241605241605242    0.153153153153153
R05149_11    0    0    0    0    0.0372093023255814    0.0772093023255814    0.111627906976744    0.273488372093023    0.151627906976744
R05151_1-2-5    0    0    0    0    0.0262235096813539    0.0794328403720079    0.0991004726330233    0.27656654977893    0.1468211617624б
```

Normal text file   length : 271673   lines : 1191   Ln : 1   Col : 1   Sel : 0 | 0   Dos\Windows   UTF-8 w/o BOM   INS

Figure 3. Example of text file containing EmsB peak and normalized fluorescent intensity values.

VII. Euclidean distance calculation, dendrogram and examples for training

Similarities or differences between isolates is tested by the Euclidean distance calculation. Thanks to Knapp and co-workers (2007), two samples are considered similar if the distance between the two samples is under 0.08.

The relationships between samples are represented by generating dendrograms of distance, using the Unweighted Pair Group Method with Arithmetic mean (UPGMA), which is a simple agglomerative hierarchical clustering method, based on pairwise similarity between units. The algorithm allows us to build a phylogenetic tree reflecting the structure present in a pairwise similarity matrix. Here the tree is considered as a dendrogram, not as a phylogenetic tree, because the homozygosis or heterozygosis origins of the EmsB loci are unknown. For each successive iteration the nearest two clusters are combined into a higher-level cluster. The arithmetic average distance between two isolates is calculated for each cluster constituted.

Because of the UPGMA method, the representation of the relationships between samples could change according to the isolates included in the model.

Three analyses are described here with R scripts:

1. **Generate a distance matrix**,
2. **Generate a dendrogram**
3. **Compare one isolate to the data collection**

For training, download the file "EmsB_text_file_example.txt" and copy the script on Tinn-R or R-studio (in a new script file) and use R software for analysis.

1. Generate a distance matrix

```
### Distance matrix on normalized EmsB profiles

#read the table with EmsB data
bdd2<-read.table("EmsB_text_file_example.txt", header=T, row.names=1)

#to see the first line of the table
head(bdd2)

#calculation of Euclidean distance amongst samples
dist2<-dist(bdd2, method="euclidian")
head(dist2)

#to obtain the distance matrix
as.matrix(dist2)

#save as a text file
write.table(as.matrix(dist2),file="distances_samples.txt", sep="\t", dec=",")
```

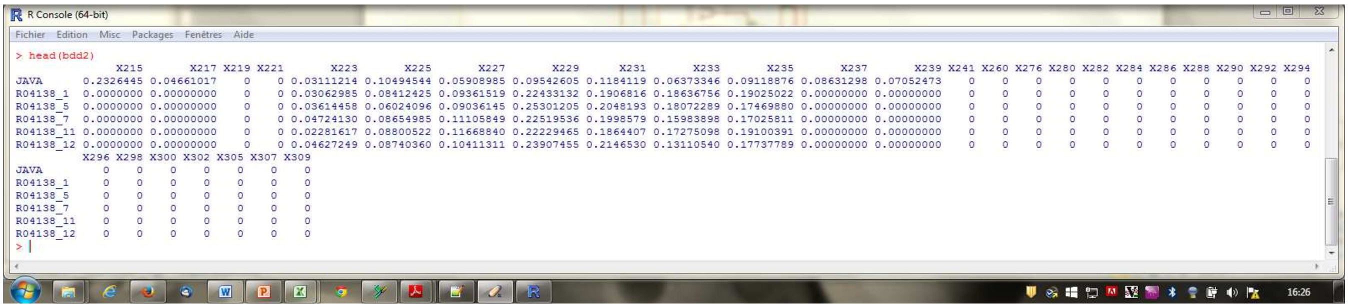

```
R Console (64-bit)
Fichier  Edition  Misc  Packages  Fenêtres  Aide
> head(bdd2)
                X215       X217 X219 X221       X223       X225       X227       X229      X231       X233       X235       X237       X239 X241 X260 X276 X280 X282 X284 X286 X288 X290 X292 X294
JAVA       0.2326445 0.04661017    0    0 0.03111214 0.10494544 0.05908985 0.09542605 0.1184119 0.06373346 0.09118876 0.08631298 0.07052473    0    0    0    0    0    0    0    0    0    0    0
R04138_1   0.0000000 0.00000000    0    0 0.03062985 0.08412425 0.09361519 0.22433132 0.1906816 0.18636756 0.19025022 0.00000000 0.00000000    0    0    0    0    0    0    0    0    0    0    0
R04138_5   0.0000000 0.00000000    0    0 0.03614458 0.06024096 0.09036145 0.25301205 0.2048193 0.18072289 0.17469880 0.00000000 0.00000000    0    0    0    0    0    0    0    0    0    0    0
R04138_7   0.0000000 0.00000000    0    0 0.04724130 0.08654985 0.11105849 0.22519536 0.1998579 0.15983898 0.17025811 0.00000000 0.00000000    0    0    0    0    0    0    0    0    0    0    0
R04138_11  0.0000000 0.00000000    0    0 0.02281617 0.08800522 0.11668840 0.22229465 0.1864407 0.17275098 0.19100391 0.00000000 0.00000000    0    0    0    0    0    0    0    0    0    0    0
R04138_12  0.0000000 0.00000000    0    0 0.04627249 0.08740360 0.10411311 0.23907455 0.2146530 0.13110540 0.17737789 0.00000000 0.00000000    0    0    0    0    0    0    0    0    0    0    0
          X296 X298 X300 X302 X305 X307 X309
JAVA         0    0    0    0    0    0    0
R04138_1     0    0    0    0    0    0    0
R04138_5     0    0    0    0    0    0    0
R04138_7     0    0    0    0    0    0    0
R04138_11    0    0    0    0    0    0    0
R04138_12    0    0    0    0    0    0    0
>
```

Figure 4. Print screen of the "head (bdd2)" control on R.

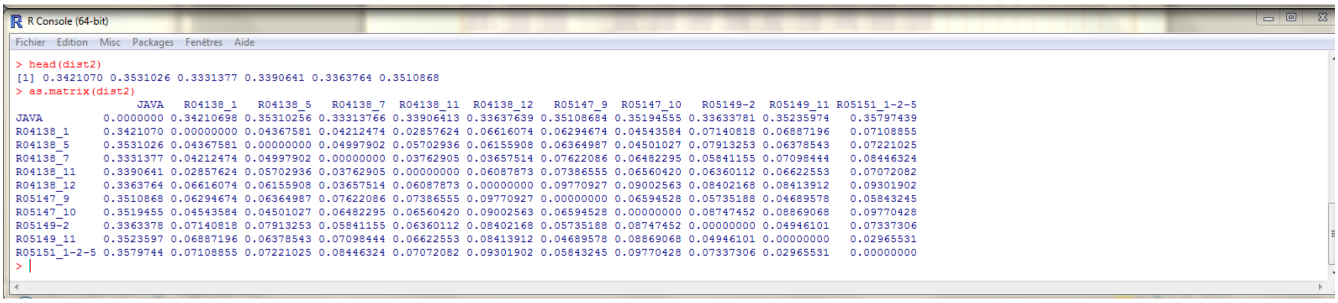

```
R Console (64-bit)
Fichier  Edition  Misc  Packages  Fenêtres  Aide
> head(dist2)
[1] 0.3421070 0.3531026 0.3331377 0.3390641 0.3363764 0.3510868
> as.matrix(dist2)
                JAVA   R04138_1   R04138_5   R04138_7  R04138_11  R04138_12   R05147_9  R05147_10  R05149-2  R05149_11 R05151_1-2-5
JAVA       0.0000000 0.34210698 0.35310256 0.33313766 0.33906413 0.33637639 0.35108684 0.35194555 0.33633781 0.35235974   0.35797439
R04138_1   0.3421070 0.00000000 0.04367581 0.04212474 0.02857624 0.06616074 0.06294674 0.04543584 0.07140818 0.06887196   0.07108855
R04138_5   0.3531026 0.04367581 0.00000000 0.04997902 0.05702936 0.06155908 0.06364987 0.04501027 0.07913253 0.06378543   0.07221025
R04138_7   0.3331377 0.04212474 0.04997902 0.00000000 0.03762905 0.03657514 0.07622086 0.06482295 0.05841155 0.07098444   0.08446324
R04138_11  0.3390641 0.02857624 0.05702936 0.03762905 0.00000000 0.06087873 0.07386555 0.06560420 0.06360112 0.06622553   0.07072082
R04138_12  0.3363764 0.06616074 0.06155908 0.03657514 0.06087873 0.00000000 0.09770927 0.09002563 0.08402168 0.08413912   0.09301902
R05147_9   0.3510868 0.06294674 0.06364987 0.07622086 0.07386555 0.09770927 0.00000000 0.06594528 0.05735188 0.04689578   0.05843245
R05147_10  0.3519455 0.04543584 0.04501027 0.06482295 0.06560420 0.09002563 0.06594528 0.00000000 0.08747452 0.08869068   0.09770428
R05149-2   0.3363378 0.07140818 0.07913253 0.05841155 0.06360112 0.08402168 0.05735188 0.08747452 0.00000000 0.04946101   0.07337306
R05149_11  0.3523597 0.06887196 0.06378543 0.07098444 0.06622553 0.08413912 0.04689578 0.08869068 0.04946101 0.00000000   0.02965531
R05151_1-2-5 0.3579744 0.07108855 0.07221025 0.08446324 0.07072082 0.09301902 0.05843245 0.09770428 0.07337306 0.02965531   0.00000000
>
```

Figure 5. Print screen of the "as.matrix (distance2)" control on R, the distance matrix

2. Generate a dendrogram

```
### Dendrogram building on normalized profiles

#read the table with EmsB data
bdd2<-read.table("EmsB_text_file_example.txt", header=T, row.names=1)

#hierarchical clustering analysis with average method
clust2<-hclust(dist2, method="average")

#obtain the dendrogram with Euclidean distance
plot(clust2,cex=0.5, main="ratio somme", hang=-1)

## Test the robustness of the clusters

#load the 'pvclust' package and perform 1000 bootstrap resampling
pv2<-pvclust(t(bdd2), method.hclust="average", method.dist="euclidian", nboot=1000)

#obtain the dendrogram with Euclidean distance
plot(pv2,cex=0.7, main="ratio somme", hang=-1)
```

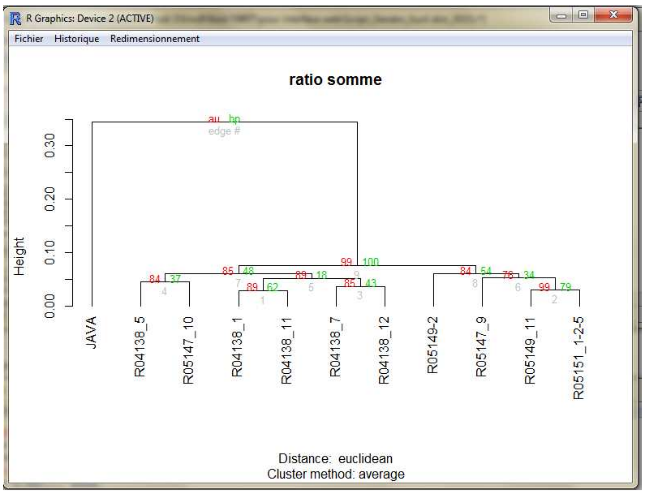

Figure 6. Print screen of the dendrogram generated associated with the bootstrap analysis (pvclust) (au for approx. unbiased value, bp for bootstrap).

3. Compare one isolate to the data collection

For training download the files "R05149_2.txt" and "EmsB_text_file_example.txt".

```
## Comparison between a single isolate and a data collection

#read the table with EmsB data containing the data collection
bdd2<-read.table("EmsB_text_file_example.txt", header=T)

#read the table with EmsB data containing the single isolate to test
ind.supp<-read.table("R05149_2.txt", header=T)

#to see the first line of the table
head(bdd2)

head(ind.supp)

## Euclidean distance calculation between the tested sample "R05149_2" and the collection samples
"EmsB_text_file_example"

bdd3=bdd2[,-1] #colomn with sample names removed

#same for " R05149_2"
ind.supp2 <- ind.supp[,-1]
fac <- function(x) {sqrt(sum((ind.supp2[1,]-bdd3[x,])^2))}
dist.eucl <- sapply(1:length(bdd3[,1]),fac)
bdd2$dist.eucl <- dist.eucl
head(bdd2)

#classification of samples according to the distance with the tested samples
tab.order <- bdd2[order(dist.eucl),]

#extraction of table lines where the "dist.eucl" < 0.08
ind.proches <- tab.order[tab.order$dist.eucl<0.08,]
ind.proches[1,1]

#sample names and Euclidean distance for which " dist.eucl " with the tested sample is <0.08
ind.proches[,which(colnames(ind.proches) %in% c("a", "dist.eucl"))]

#to get the top 5 of the samples the most similar to the tested sample
ind.proches[1:5,which(colnames(ind.proches) %in% c("a", "dist.eucl"))]

#find a sample and its dist.eucl with the tested sample for ex. " R05149_11 "
ind.proches[ind.proches$a=="R05149_11",which(colnames(ind.proches) %in% c("a", "dist.eucl"))]
```

```
#to get the top 5 of the samples the most similar to the tested sample
```

➪ *Result:*

```
> ind.proches[1:5,which(colnames(ind.proches) %in% c("a", "dist.eucl"))]
                    a   dist.eucl
9     R05149_2      0.00000000
10    R05149_11     0.04946101
7     R05147_9      0.05735188
4     R04138_7      0.05841155
5     R04138_11     0.06360112
```

➪ *Interpretation*: the list of the 5 closest samples to R05149_2 isolate. All are under 0.08 in distance and are thus considered similar to the tested sample.

```
#find a sample and its dist.eucl with the tested sample for ex. " R05149_11 "
```

➪ *Result:*

```
> ind.proches[ind.proches$a=="R05149_11",which(colnames(ind.proches) %in% c("a", "dist.eucl"))]

           a   dist.eucl

10 R05149_11 0.04946101
```

➪ *Interpretation*: sample R04149_11 is distant from R04149_2 with a distance of 0.049. They are considered similar to each other.

References

1. Valot B, Knapp J, Umhang G, Grenouillet F, Millon L. Genomic characterization of EmsB microsatellite loci in *Echinococcus multilocularis*. Infect Genet Evol. 2015;32: 338–341.

2. Bart JM, Knapp J, Gottstein B, El-Garch F, Giraudoux P, Glowatzki ML, et al. EmsB, a tandem repeated multi-loci microsatellite, new tool to investigate the genetic diversity of *Echinococcus multilocularis*. Infect Genet Evol. 2006;6: 390–400.

3. Knapp J, Bart JM, Glowatzki ML, Ito A, Gerard S, Maillard S, et al. Assessment of use of microsatellite polymorphism analysis for improving spatial distribution tracking of *Echinococcus multilocularis*. J Clin Microbiol. 2007;45: 2943–2950.

4. Knapp J, Guislain M-H, Bart JM, Raoul F, Gottstein B, Giraudoux P, et al. Genetic diversity of *Echinococcus multilocularis* on a local scale. Infect Genet Evol. 2008;8: 367–373.

5. Casulli A, Bart JM, Knapp J, La Rosa G, Dusher G, Gottstein B, et al. Multi-locus microsatellite analysis supports the hypothesis of an autochthonous focus of *Echinococcus multilocularis* in northern Italy. Int J Parasitol. 2009;39: 837–842.

6. Knapp J, Bart J-M, Giraudoux P, Glowatzki M-L, Breyer I, Raoul F, et al. Genetic diversity of the cestode *Echinococcus multilocularis* in red foxes at a continental scale in Europe. PLoS Negl Trop Dis. 2009;3: e452.

7. Knapp J, Staebler S, Bart JM, Stien A, Yoccoz NG, Drögemüller C, et al. *Echinococcus multilocularis* in Svalbard, Norway: microsatellite genotyping to investigate the origin of a highly focal contamination. Infect Genet Evol. 2012;12: 1270–1274.

8. Umhang G, Knapp J, Hormaz V, Raoul F, Boué F. Using the genetics of *Echinococcus multilocularis* to trace the history of expansion from an endemic area. Infect Genet Evol. 2014;22: 142–149.
